# Supplementary material for: High-resolution in situ structures of mammalian respiratory supercomplexes
Source: Nature. 2024 May 29;631(8019):232–9. doi: 10.1038/s41586-024-07488-9 (PMC11222160; doi:10.1038/s41586-024-07488-9)
Supplement: Supplementary file 1 — Supplementary Figs. 1–13 and Tables 1–8. [file 41586_2024_7488_MOESM1_ESM.pdf]

nature portfolio

https://doi.org/10.1038/s41586-024-07488-9

Supplementary information

High-resolution in situ structures of mammalian respiratory supercomplexes

In the format provided by the authors and unedited

Supplementary information

High-resolution *In-situ* Structures of Mammalian Respiratory Supercomplexes

Wan Zheng[1,2], Pengxin Chai[2], Jiapeng Zhu[1#], Kai Zhang[2#]

1. School of Medicine & Holistic Integrative Medicine, Nanjing University of Chinese Medicine, Nanjing, 210023, China
2. Department of Molecular Biophysics and Biochemistry, Yale University, New Haven, CT, 06511, USA

Correspondence: zhujiapeng@hotmail.com and jack.zhang@yale.edu

Table of Contents

Supplementary Figures 1

Supplementary Fig. 1 Cryo-ET data processing flowchart. 1

Supplementary Fig. 2 Schematic of the workflow for multi-level refinement and focused classification of type-A supercomplex. 2

Supplementary Fig. 3 Schematic of the workflow for multi-level refinement of type-B supercomplex....................4

Supplementary Fig. 4 Schematic of the workflow for multi-level refinement of type-O supercomplex....................5

Supplementary Fig. 5 Schematic of the workflow for multi-level refinement of type-X supercomplex....................6

Supplementary Fig. 6 Representative densities in CIII of all types of residues demonstrate high-resolution features enabling unambiguous rotamer determination. 7

Supplementary Fig. 7 Atomic-level details of interaction interface among individual complexes. ...8

Supplementary Fig. 8 Architecture and symmetry of in-situ supercomplex structures and interface comparison with the in-vitro supercomplex $I_1III_2IV_1$....................9

Supplementary Fig. 9 CIV' partially blocks the space between the Q-sites of CI and CIII in type-B supercomplex....................10

Supplementary Fig. 10 Localization of NDUFA4 in the in-situ respiratory supercomplex....................10

Supplementary Fig. 11 Analysis of the hallmarks of the four distinct Q-occupied states....................11

Supplementary Fig. 12 | Focused 3D classification targeting the Q-site identifies 7 major intermediate classes.....................12

Supplementary Fig. 13 Comparison conformational changes of CI ND6-TMH4 during active/deactive transition across different species....................13

Supplementary Tables....................14

Supplementary Table 1. Cryo-EM data collection, refinement and validation statistics 14

Supplementary Table 2. Cryo-EM data collection, refinement and validation statistics 15

Supplementary Table 3. Cryo-EM data collection, refinement and validation statistics 16

Supplementary Table 4. Cryo-EM data collection, refinement and validation statistics 17

Supplementary Table 5. Cryo-EM data collection, refinement and validation statistics 18

Supplementary Table 6. Cryo-EM data collection, refinement and validation statistics 19

Supplementary Table 7. Isoform identification for CIV subunits. 20

Supplementary Table 8. Analysis of "hallmarks" across different classes for combined datasets. ...21

Supplementary Figures

Supplementary Fig. 1 Cryo-ET data processing flowchart.

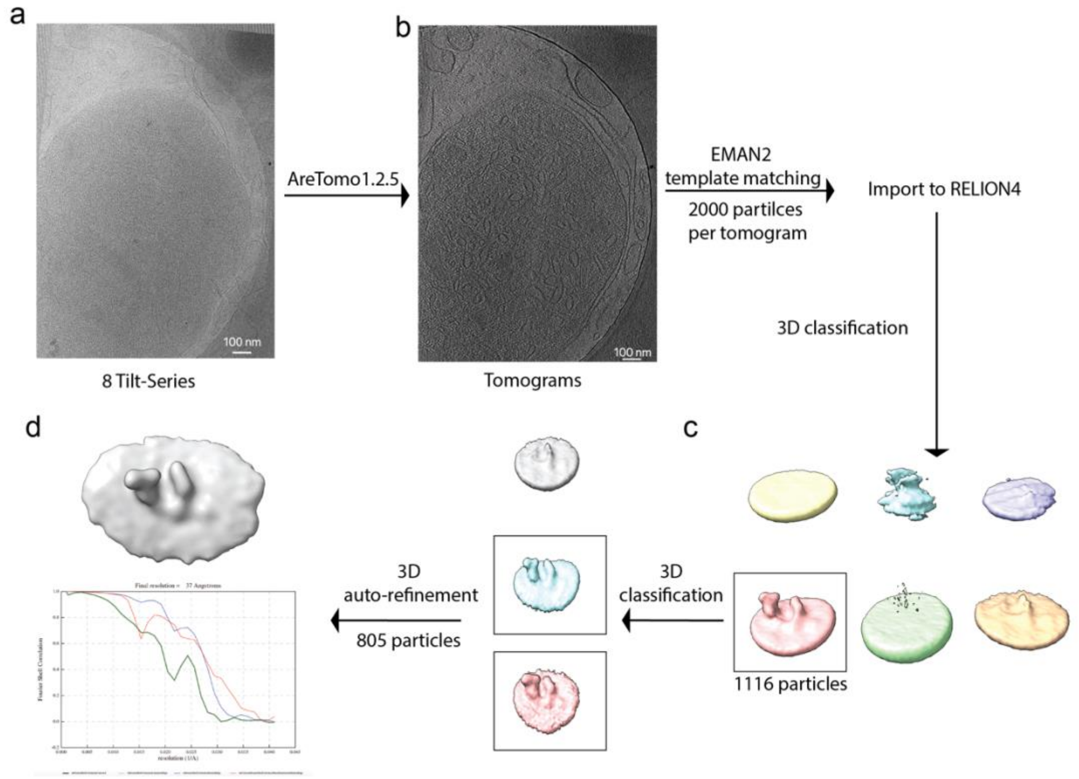

Supplementary Fig. 1 | Cryo-ET data processing flowchart.

a, Representative zero tilt image from 8 tilt series. **b,** Representative slice of the reconstructed tomogram. **c,** Intermediate results of the sub-volume averaging by *Relion 4.0*. **d,** Final reconstructed map and FSC curve of the type-A supercomplex.

Supplementary Fig. 2 Schematic of the workflow for multi-level refinement and focused classification of type-A supercomplex.

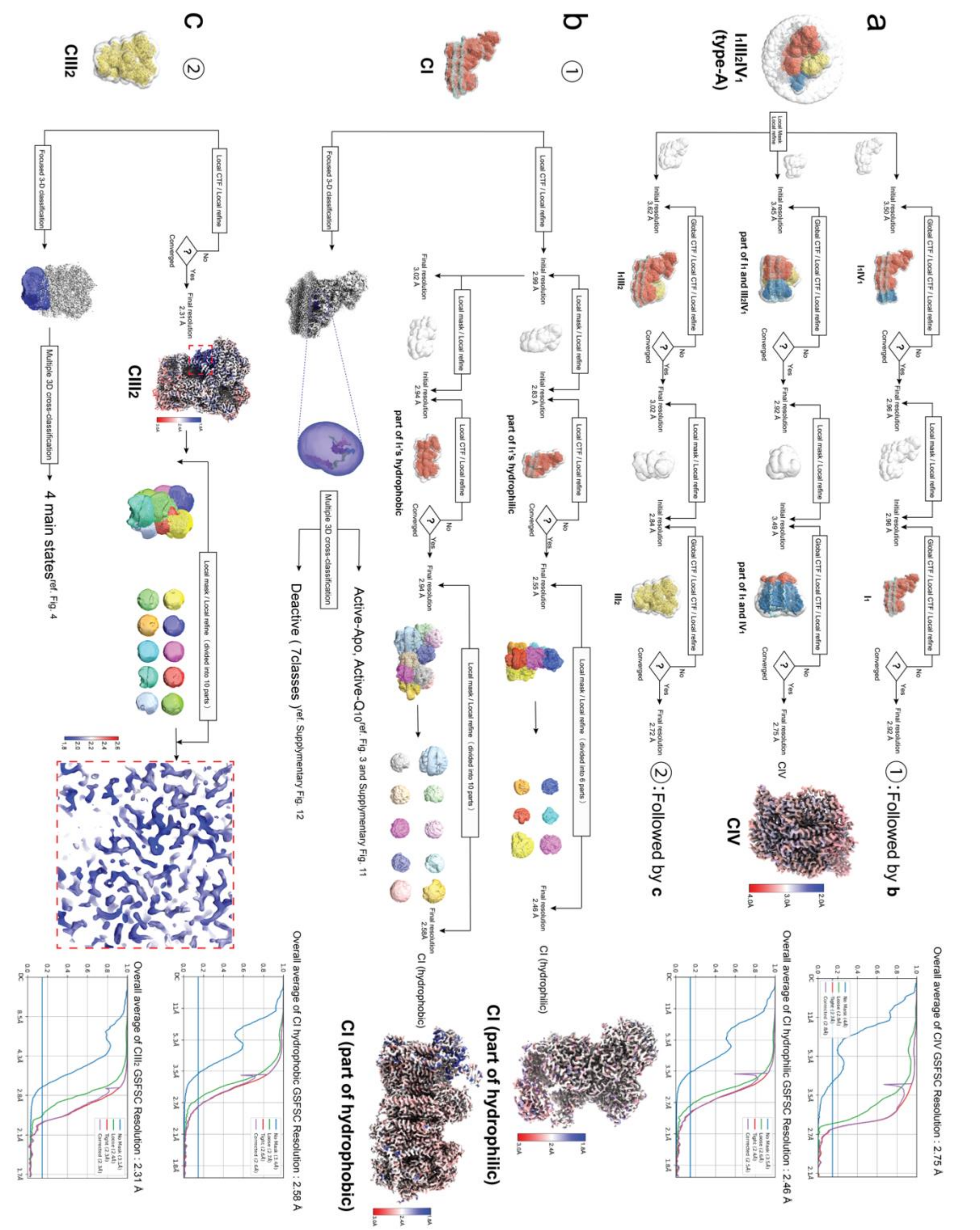

Supplementary Fig. 2 | Schematic of the workflow for multi-level refinement and focused classification of type-A supercomplex.

a, Multiple cycles of local refinement that generated maps of CI (red), CIII (yellow), CIV (blue). The masks are indicated by transparent white density. The initial type-A map is derived from

the method described in Extended Data Fig. 1, with an average resolution of 3.5Å. Three masks were applied to CI, CIII and CIV for local refinement, followed by global CTF, local CTF, and local refinement iteratively until converged, which generated CI, CIII and CIV maps at 2.96 Å, 3.49 Å and 2.84 Å, respectively. CIV was locally refined using a single mask covering the whole CIV, whereas CI and CIII were divided into more local regions for further improvement. **b,** Multi-level local refinement of CI and focused classification of Q_{10} region (part ①). CI was first divided into the hydrophilic and hydrophobic regions and refined using similar approaches as described in (**a**), which generated maps in the two regions at 2.46 Å and 2.58 Å, respectively. Upon this, the hydrophilic and hydrophobic regions are further subdivided into six and eight smaller sections (indicated by colored masks) for further local refinement. The lower section focuses on the Q10 region (purple mask) for 3D classification**. c,** Multi-level local refinement of CIII and focused classification of the Rieske domain (part ②). CIII was first locally refined to 2.31 Å resolution using a masking covering the whole CIII and then divided into 10 local regions using the similar approaches for CI refinement.

Supplementary Fig. 3 Schematic of the workflow for multi-level refinement of type-B supercomplex.

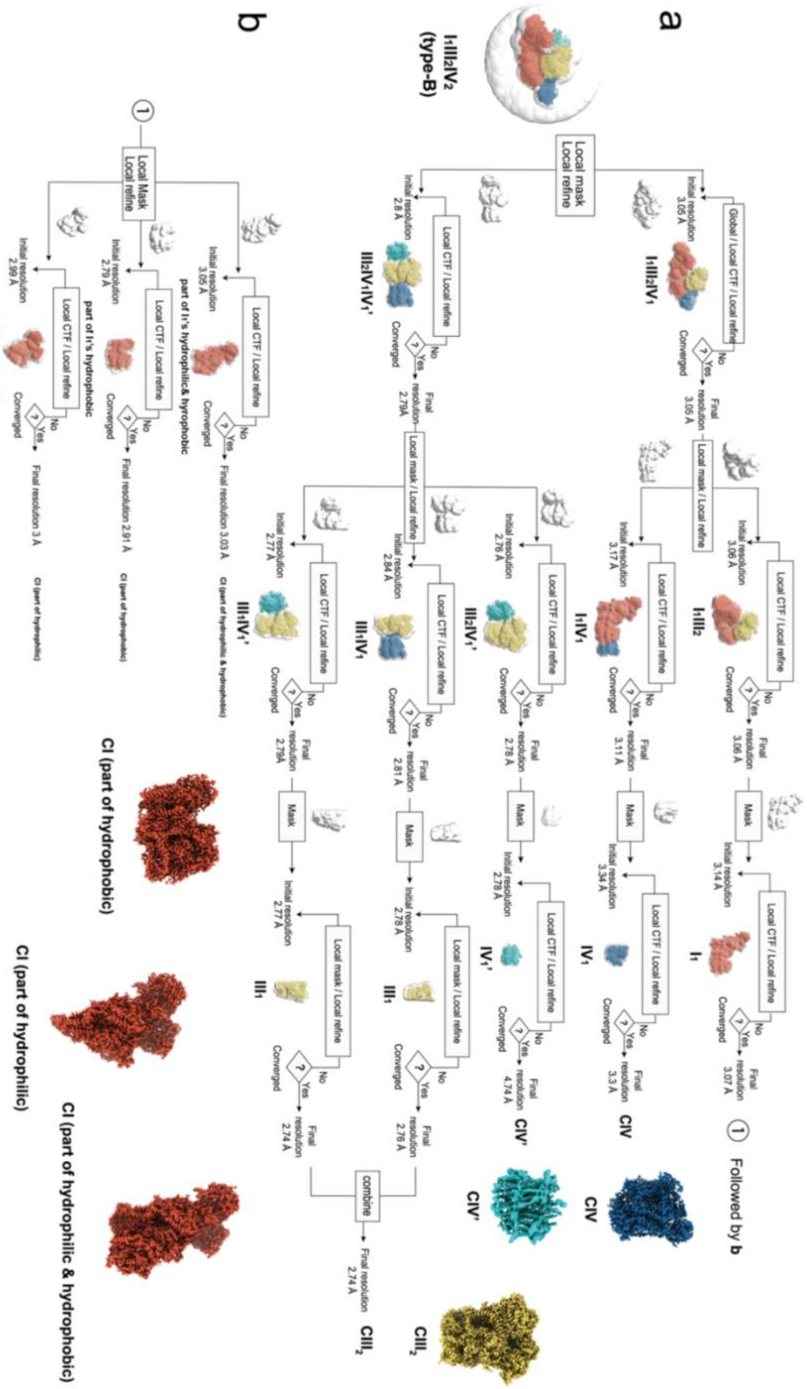

Supplementary Fig. 3 | Schematic of the workflow for multi-level refinement of type-B supercomplex.

The refinement process for type-B is similar to that of type-A. Initially, a map with an average resolution of 3.5 Å is obtained from Extended Data Fig. 2, followed by a stepwise approach to local masking and refinement, starting from the largest to the smallest components. In this map, red, yellow, dark blue and cyan denotes CI, $CIII_2$, CIV, and CIV', respectively.

Supplementary Fig. 4 Schematic of the workflow for multi-level refinement of type-O supercomplex.

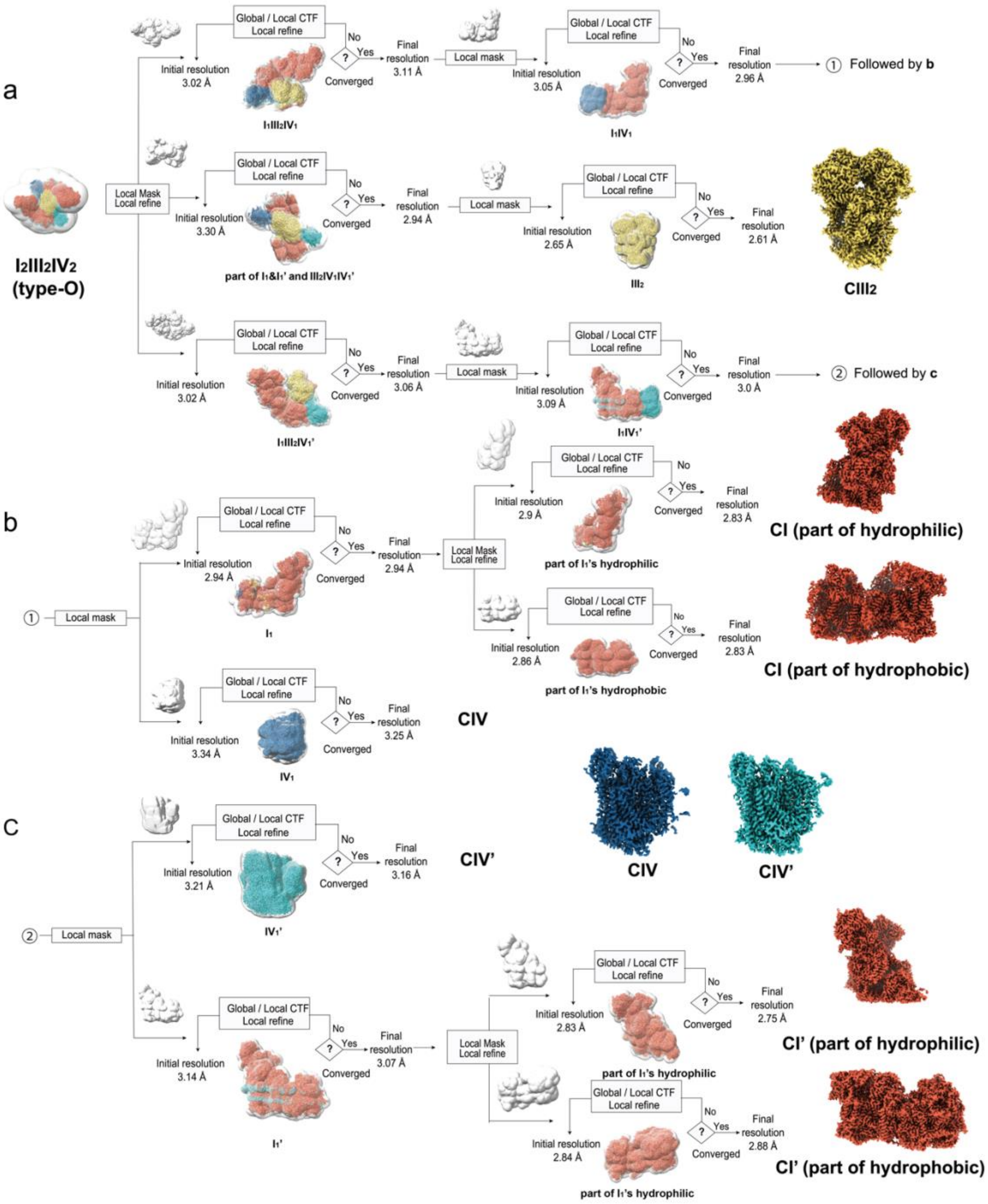

Supplementary Fig. 4 | Schematic of the workflow for multi-level refinement of type-O supercomplex.

The refinement process for type-O is similar to that of type-A. Initially, a map with an average resolution of 3.5 Å is obtained from Extended Data Fig. 2, followed by a stepwise approach to local masking and refinement, starting from the largest to the smallest components. In this map, red, yellow, dark blue and cyan denotes CI, $CIII_2$, CIV, and CIV', respectively.

Supplementary Fig. 5 Schematic of the workflow for multi-level refinement of type-X supercomplex.

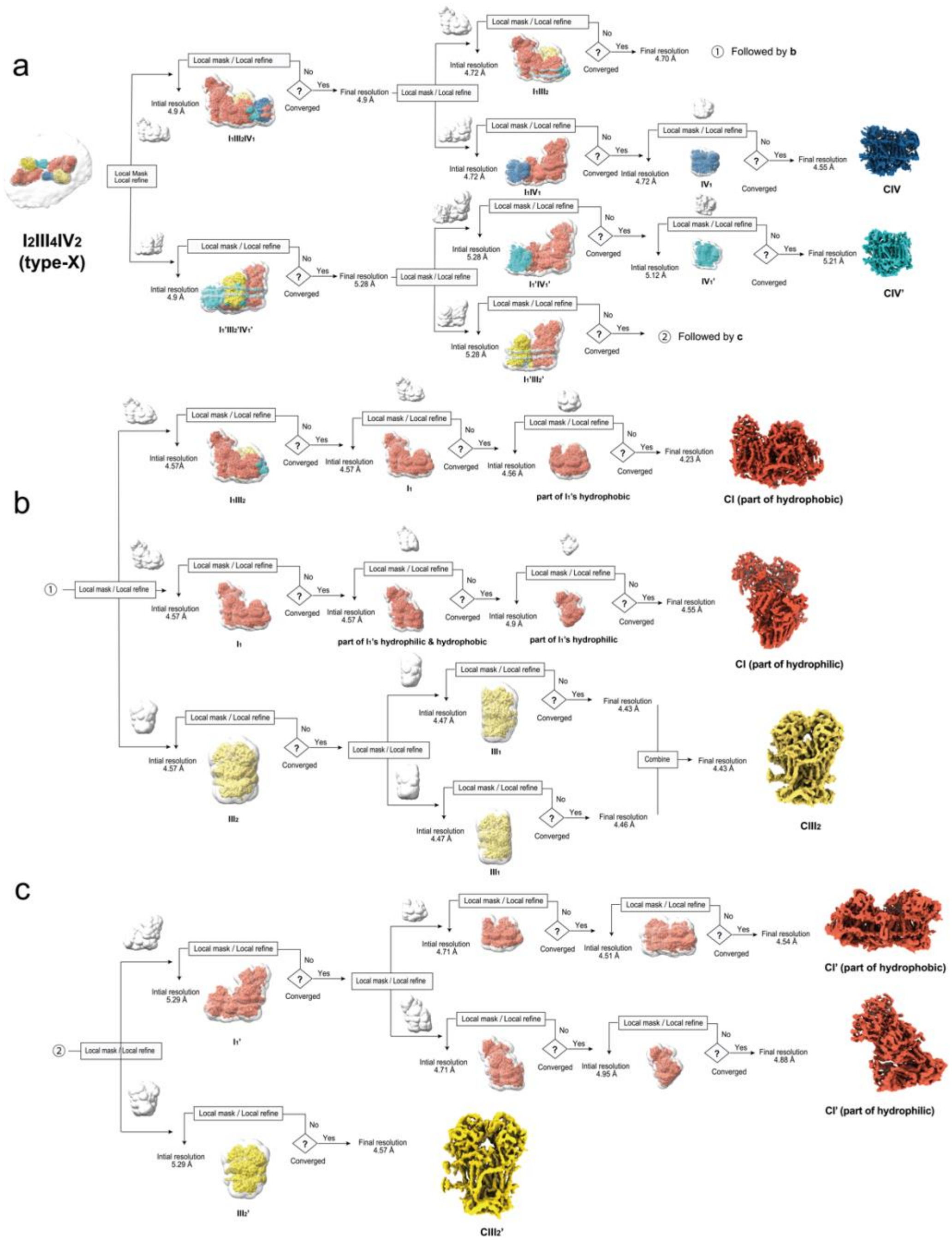

Supplementary Fig. 5 | Schematic of the workflow for multi-level refinement of type-X supercomplex.

The refinement process for type-X is similar to that of type-A. Initially, a map with an average resolution of 3.5 Å is obtained from Extended Data Fig. 2, followed by a stepwise approach to local masking and refinement, starting from the largest to the smallest components. In this map, red, yellow, dark blue and cyan denotes CI, $CIII_2$, CIV, and CIV', respectively.

Supplementary Fig. 6 Representative densities in CIII of all types of residues demonstrate high-resolution features enabling unambiguous rotamer determination.

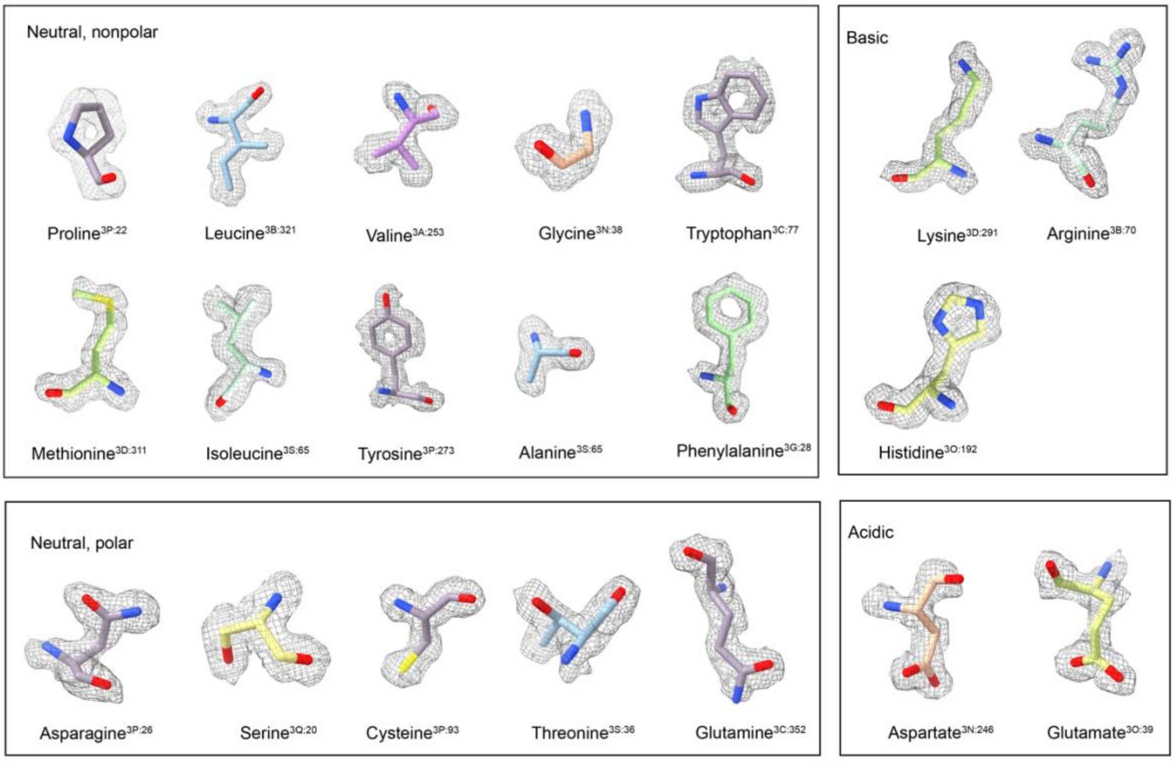

Supplementary Fig. 6 | Representative densities in CIII of all types of residues demonstrate high-resolution features enabling unambiguous rotamer determination. Notably, the distinct holes in the six-element rings of tryptophan, phenylalanine, and tyrosine are prominently visible.

Supplementary Fig. 7 Atomic-level details of interaction interface among individual complexes.

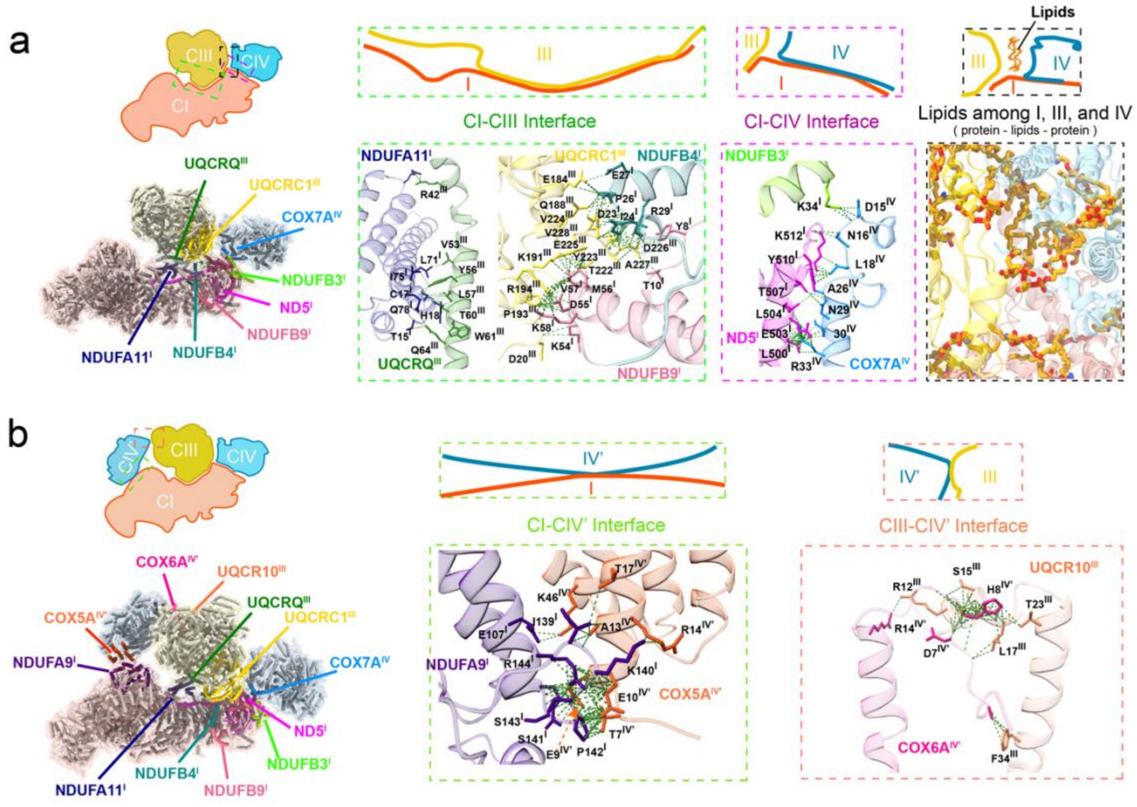

Supplementary Fig. 7 | Atomic-level details of interaction interface among individual complexes.

a,b, The interactions involve both protein-protein and protein-lipids-protein interactions (Supplementary Video 5). In particular, no direct protein-protein interactions are observed between CIII and CIV. Dashed lines indicate close atomic contacts with van der Waals (VDW) overlap greater than 0.4 Å, analyzed in ChimeraX.

Supplementary Fig. 8 Architecture and symmetry of in-situ supercomplex structures and interface comparison with the in-vitro supercomplex $I_1III_2IV_1$.

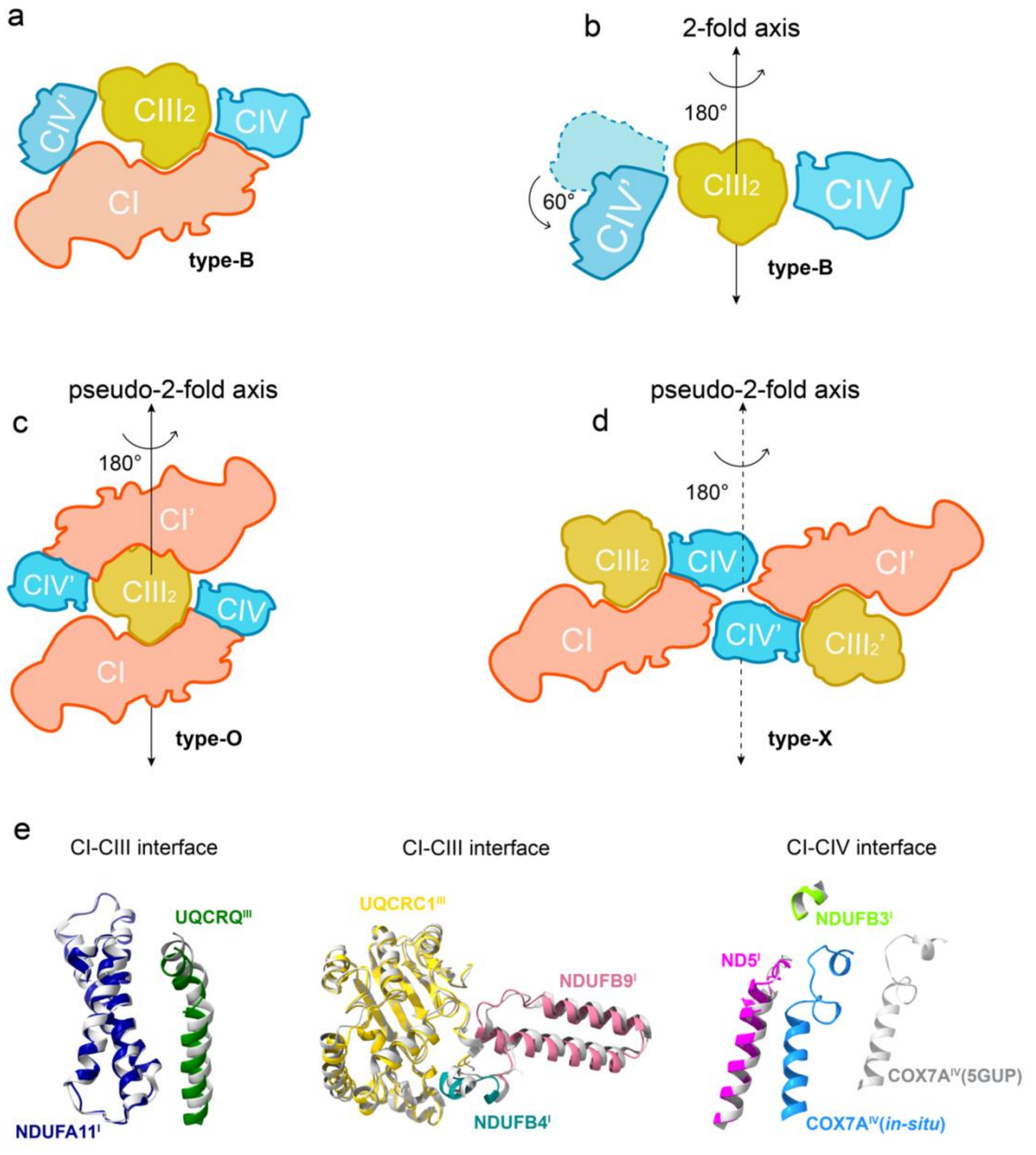

Supplementary Fig. 8 | Architecture and symmetry of *in-situ* supercomplex structures and interface comparison with the *in-vitro* supercomplex $I_1III_2IV_1$.

a,b, Type B supercomplex exhibits asymmetry in its two CIVs along the 2-fold axis of the CIII dimer. **c,d,** Both type O and type X supercomplexes display a pseudo-2-fold axis due to membrane curvature-induced distortion. **e,** Interface comparison between individual complexes CI-CIII and CI-CV in our *in-situ* type A supercomplex (colored) and the *in-vitro* supercomplex $I_1III_2IV_1$ (white, PDB code: 5GUP) reveals evident differences.

Supplementary Fig. 9 CIV' partially blocks the space between the Q-sites of CI and CIII in type-B supercomplex.

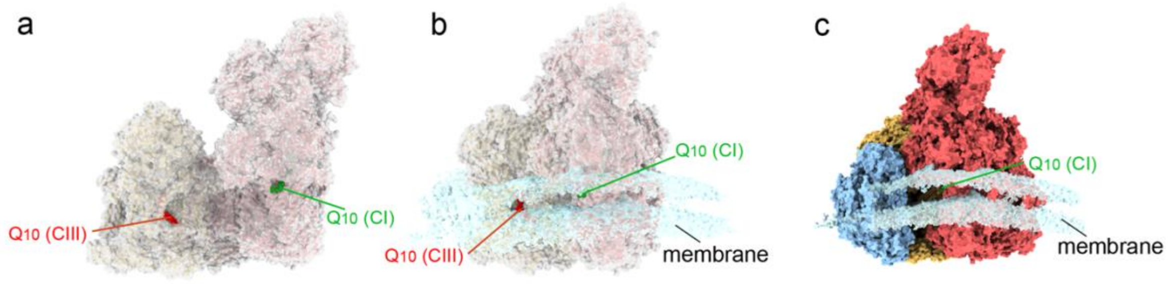

Supplementary Fig. 9 | CIV' partially blocks the space between the Q-sites of CI and CIII in type-B supercomplex.

a, Q-sites of CI and CIII in type-A supercomplex. **b,** Q-sites of CI and CIII in type-A supercomplex with the membrane shown. **c,** CIV' partially blocks the space between the Q-sites of CI and CIII in type-B supercomplex, forming an architecture that could confine the free diffusion of Q_{10} and facilitate the Q-channeling.

Supplementary Fig. 10 Localization of NDUFA4 in the in-situ respiratory supercomplex.

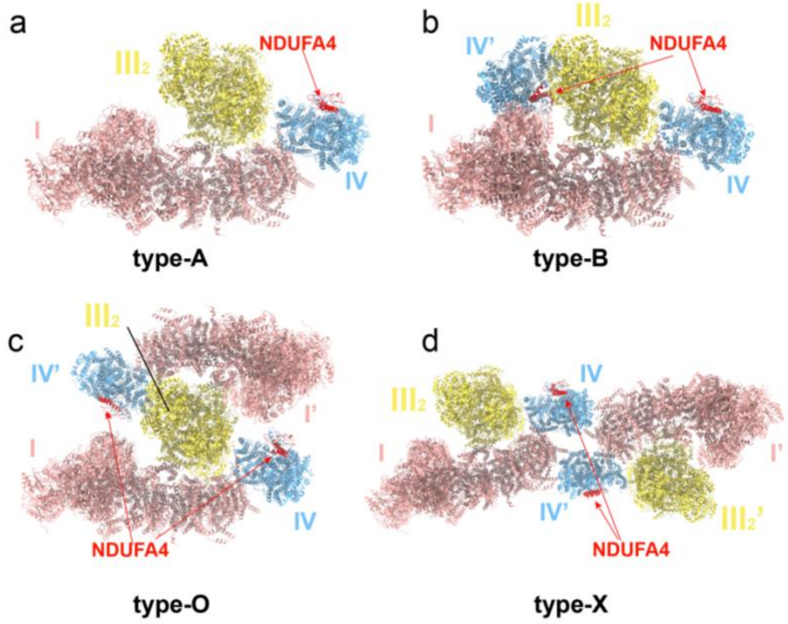

Supplementary Fig. 10 | Localization of NDUFA4 in the *in-situ* respiratory supercomplex. **a, b, c,d,** Localization of NDUFA4 (red) in type-A, B, O, and X supercomplexes. Complex I is shown in pale red, Complex III_2 in yellow, and Complex IV in blue. NDUFA4 is positioned on CIV, distant from CI in all supercomplex types.

Supplementary Fig. 11 Analysis of the hallmarks of the four distinct Q-occupied states.

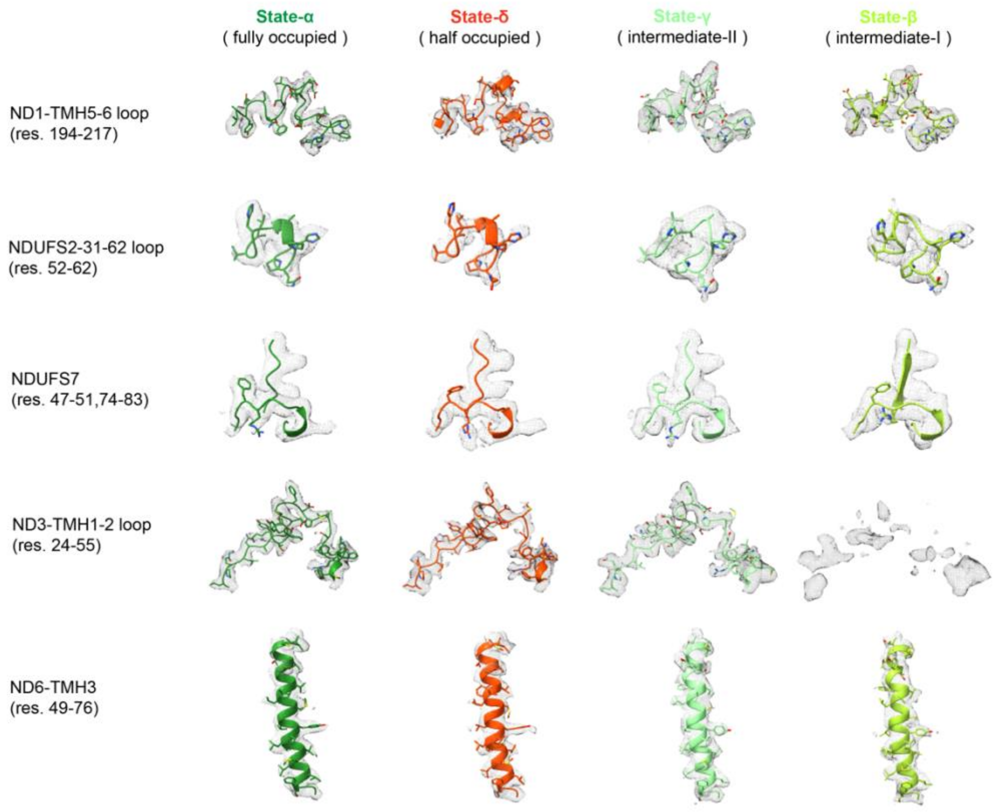

Supplementary Fig. 11 | Analysis of the hallmarks of the four distinct Q-occupied states. State-α, -γ, -δ exhibit standard active features, while State-β show partial deactive features, suggesting these conformational changes may be required for the intermediate states during the reaction.

Supplementary Fig. 12 | Focused 3D classification targeting the Q-site identifies 7 major intermediate classes.

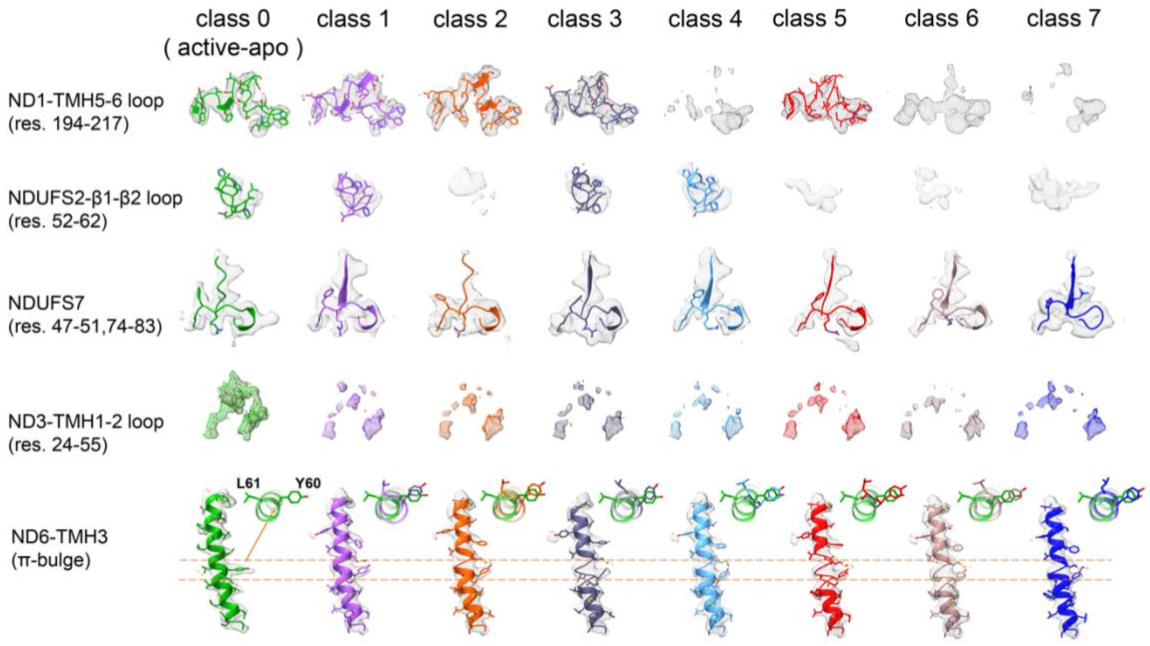

Supplementary Fig. 12 | Focused 3D classification targeting the Q-site identifies 7 major intermediate classes.

Two extreme classes (class-0 and 7) fully conform to all the hallmarks of the active and deactive states, respectively. Other classes exhibit only a subset of the hallmarks associated with the deactive state, suggesting that they represent various stages in the transition between the two extreme states. Within ND6-TMH3, the active conformation is characterized by an α-helix structure, whereas the deactive state is distinguished by a π-bulge configuration. Notably, the most significant distinction involves the displacement of residue Y60 and Y61 in the deactive state compared to its position in the active conformation.

Supplementary Fig. 13 Comparison conformational changes of CI ND6-TMH4 during active/deactive transition across different species.

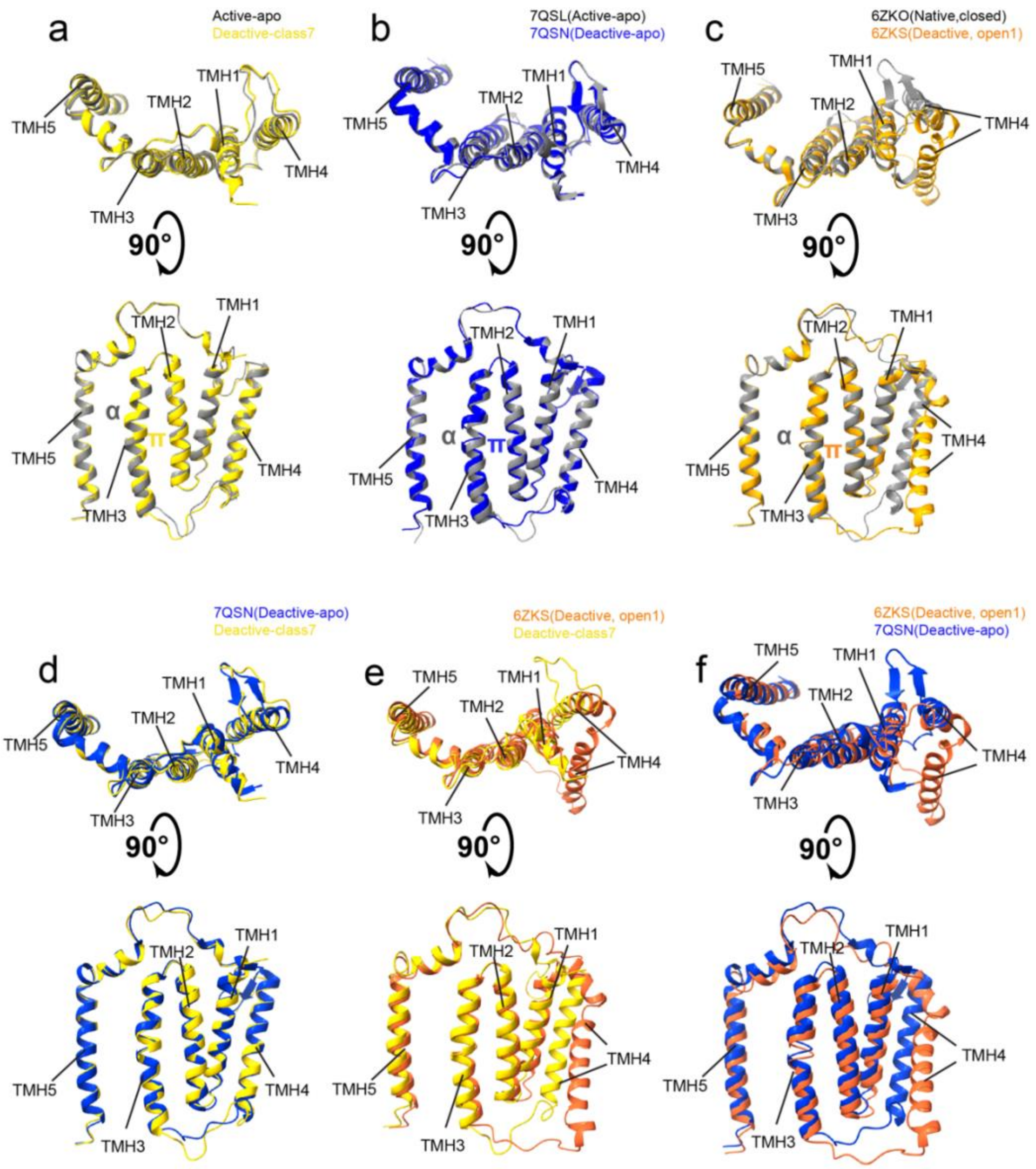

Supplementary Fig. 13 | Comparison conformational changes of CI ND6-TMH4 during active/deactive transition across different species.

a-**c,** Comparison of CI ND6-TMH4 between active and deactive states from different species. The active states are shown in gray: active-apo (this work, porcine), active-apo (PDB ID: 7QSL, bovine), and native-closed (PDB ID: 6ZKO, ovine). The deactive states are shown in color: deactive-class7 (this work, porcine), deactive-apo (PDB ID: 7QSN, bovine), and deactive-open1 (PDB ID: 6ZKS, ovine). The relocation of TMH4 is only observed in ovine. **d-f,** Comparison of CI ND6-TMH4 deactive states between porcine and bovine **(d)**, porcine and ovine **(e)**, and bovine and ovine **(f)**. Porcine is shown in yellow, bovine blue and ovine orange.

Supplementary Tables

Supplementary Table 1. Cryo-EM data collection, refinement and validation statistics

	In-situ structure of supercomplex in respiratory chain （ $I_1III_2IV_1$, type-A） (EMD-42226) (8UGI)	*In-situ* structure of supercomplex in respiratory chain ($I_1III_2IV_2$, type-B) (EMD-42227) (8UGJ)	*In-situ* structure of supercomplex in respiratory chain ($I_2III_2IV_2$, type-O) （EMD-42230） （8UGN）	*In-situ* structure of supercomplex in respiratory chain （ $I_2III_4IV_2$, type-X） （EMD-42233） （8UGR）
**Data collection and processing**				
Magnification	105,000	105,000	105,000	105,000
Voltage (kV)	300	300	300	300
Electron exposure (e–/Å^2)	50	50	50	50
Defocus range (μm)	-1.3 to -3.0	-1.3 to -3.0	-1.3 to -3.0	-1.3 to -3.0
Pixel size (Å)	0.832	0.832	0.832	0.832
Symmetry imposed	C1	C1	C1	C1
Initial particle images (no.)	16,785,436	16,785,436	16,785,436	16,785,436
Final particle images (no.)	561,624	175,088	70,203	70,998
Map resolution (Å)	2.5	2.6	2.7	6.5
FSC threshold	FSC = 0.143	FSC = 0.143	FSC = 0.143	FSC = 0.143
Map resolution range (Å)	1.8 – 2.9	2.2 – 3.5	2.6 – 3.3	4.5 – 10.0
**Refinement**				
Initial model used (PDB code)	*Ab-Initio*	*Ab-Initio*	*Ab-Initio*	*Ab-Initio*
Model resolution (Å)	2.5	3.1	3.1	7.3
FSC threshold	FSC = 0.5	FSC = 0.5	FSC = 0.5	FSC = 0.5
Map sharpening *B* factor (Å^2)	-50	-10.8	-14.8	-187
Model composition				
Non-hydrogen atoms	124291	134337	202921	234747
Protein residues	14242	16088	24375	28488
Ligands	MG: 2 K: 1 SF4: 6 HEC: 2 EHZ: 2 FMN: 1 HEA: 2 CUA: 1 PEK: 2 U10: 4 3PE: 27 NDP: 1 GTP: 1 ZN: 3 CDL: 15 PSC: 1 HEM: 4 MYR: 1 PGV: 15 NA: 1 PC1: 15 CU: 1 PO4: 1 FES: 4	MG: 3 K: 1 SF4: 6 HEC: 2 EHZ: 2 FMN: 1 HEA: 4 CUA: 2 PEK: 4 HEM: 4 3PE: 29 NDP: 1 GTP: 1 ZN: 3 CDL: 19 PSC: 2 MYR: 1 PGV: 30 NA: 2 PC1: 17 CU: 2 PO4: 2 FES: 4	MG: 4 K: 2 SF4: 12 HEC: 2 EHZ: 4 FMN: 2 HEA: 4 CUA: 2 PEK: 4 HEM: 4 3PE: 47 NDP: 2 GTP: 2 ZN: 4 CDL: 26 PSC: 2 MYR: 2 PGV: 30 NA: 2 PC1: 31 CU: 2 PO4: 2 FES: 6	MG: 4 K: 2 SF4: 12 HEC: 4 EHZ: 4 FMN: 2 HEA: 4 CUA: 2 PEK: 4 HEM: 8 3PE: 36 NDP: 2 GTP: 2 ZN: 4 CDL: 20 PSC: 2 MYR: 2 PGV: 30 NA: 2 PC1: 28 CU: 2 PO4: 2 FES: 8
B factors (Å^2)				
Protein	25.59	39.19	24.82	236.99
Ligand	47.03	52.38	37.32	281.40
R.m.s. deviations				
Bond lengths (Å)	0.009	0.005	0.003	0.011
Bond angles (°)	0.949	0.810	0.654	1.321
Validation				
MolProbity score	2.28	2.02	1.85	2.51
Clashscore	14.81	18.02	11.16	43.63
Poor rotamers (%)	3.05	0.30	0.28	0.82
Ramachandran plot				
Favored (%)	96.40	96.08	95.89	94.22
Allowed (%)	3.41	3.75	3.94	5.56
Disallowed (%)	0.18	0.18	0.17	0.22

Supplementary Table 2. Cryo-EM data collection, refinement and validation statistics

	High resolution *In-situ* structure of complex I in respiratory supercomplex (EMD-42143) (8UD1)	High resolution *In-situ* structure of complex III in respiratory supercomplex (EMD-42228) (8UGK)	High resolution *In-situ* structure of complex IV in respiratory supercomplex (EMD-42229) (8UGL)	*In-situ* complex I, Active-Apo (EMD-42165) (8UEO)
**Data collection and processing**				
Magnification	105,000	105,000	105,000	105,000
Voltage (kV)	300	300	300	300
Electron exposure (e–/Å^2)	50	50	50	50
Defocus range (μm)	-1.3 to -3.0	-1.3 to -3.0	-1.3 to -3.0	-1.3 to -3.0
Pixel size (Å)	0.832	0.832	0.832	0.832
Symmetry imposed	C1	C1	C1	C1
Initial particle images (no.)	16,785,436	16,785,436	16,785,436	16,785,436
Final particle images (no.)	1,029,121	1,018,624	1,072,691	47,029
Map resolution (Å)	2.1	2.1	2.9	3.8
FSC threshold	FSC = 0.143	FSC = 0.143	FSC = 0.143	FSC = 0.143
Map resolution range (Å)	1.8 – 3.2	1.8 – 2.5	2.5 – 3.6	3.5 – 4.5
**Refinement**				
Initial model used (PDB code)	*Ab-Initio*	*Ab-Initio*	*Ab-Initio*	*Ab-Initio*
Model resolution (Å)	2.5	2.4	2.9	3.9
FSC threshold	FSC = 0.5	FSC = 0.5	FSC = 0.5	FSC = 0.5
Map sharpening *B* factor (Å^2)	-78.3	-68.4	-101.7	-35.1
Model composition				
Non-hydrogen atoms	71989	35368	16572	68031
Protein residues	8287	4113	1842	8281
Ligands	FMN: 1 MG: 1 K: 1 SF4: 6 PC1: 12 EHZ: 2 GTP: 1 CDL: 6 ZN: 1 FES: 2 NDP: 1 MYR: 1 3PE: 16	FES: 2 HEC: 2 CDL: 6 HEM: 4 PC1: 3 3PE: 11	MG: 1 CDL: 1 PSC: 1 PO4: 1 PGV: 13 NA: 1 ZN: 1 HEA: 2 CUA: 1 PEK: 1 CU: 1	FMN: 1 MG: 1 K: 1 SF4: 6 PC1: 13 EHZ: 2 GTP: 1 CDL: 6 ZN: 1 FES: 2 NDP: 1 MYR: 1 3PE: 10
B factors (Å^2)				
Protein	15.66	15.01	13.11	137.90
Ligand	17.05	21.05	35.57	177.68
R.m.s. deviations				
Bond lengths (Å)	0.006	0.012	0.005	0.008
Bond angles (°)	0.818	1.241	0.900	1.159
Validation				
MolProbity score	2.29	2.00	2.22	3.03
Clashscore	13.91	16.57	14.30	32.66
Poor rotamers (%)	2.73	0.40	2.85	5.65
Ramachandran plot				
Favored (%)	96.20	95.96	96.69	92.63
Allowed (%)	3.62	3.69	3.25	7.15
Disallowed (%)	0.18	0.34	0.06	0.22

Supplementary Table 3. Cryo-EM data collection, refinement and validation statistics

	In-situ complex I with Q_{10} (State-α) (EMD-42166) (8UEP)	*In-situ* complex I with Q_{10} (State-β) (EMD-42167) (8UEQ)	*In-situ* complex I with Q_{10} (State-γ) (EMD-42168) (8UER)	*In-situ* complex I with Q_{10} (State-σ) (EMD-42176) (8UEZ)
**Data collection and processing**				
Magnification	45,000	45,000	45,000	45,000
Voltage (kV)	200	200	200	200
Electron exposure (e–/Å^2)	42	42	42	42
Defocus range (μm)	-1.0 to -3.0	-1.0 to -3.0	-1.0 to -3.0	-1.0 to -3.0
Pixel size (Å)	0.868	0.868	0.868	0.868
Symmetry imposed	C1	C1	C1	C1
Initial particle images (no.)	5,611,539	5,611,539	5,611,539	5,611,539
Final particle images (no.)	34,975	35,095	33,009	25,747
Map resolution (Å)	3.4	3.4	3.5	3.5
FSC threshold	FSC = 0.143	FSC = 0.143	FSC = 0.143	FSC = 0.143
Map resolution range (Å)	3.0 – 4.5	3.0 – 5.0	3.0 – 5.1	3.0 – 5.1
**Refinement**				
Initial model used (PDB code)	*Ab-Initio*	*Ab-Initio*	*Ab-Initio*	*Ab-Initio*
Model resolution (Å)	3.5	3.9	3.7	3.6
FSC threshold	FSC = 0.5	FSC = 0.5	FSC = 0.5	FSC = 0.5
Map sharpening *B* factor (Å^2)	-19.9	-11.4	-14.4	-6.8
Model composition				
Non-hydrogen atoms	67436	67142	67436	67334
Protein residues	8289	8289	8289	8289
Ligands	FMN: 1 MG: 1 K: 1 SF4: 6 MYR: 1 EHZ: 2 GTP: 1 CDL: 3 ZN: 1 FES: 2 U10: 1 NDP: 1 3PE: 4 PC1: 6	FMN: 1 MG: 1 K: 1 SF4: 6 MYR: 1 EHZ: 2 GTP: 1 CDL: 2 ZN: 1 FES: 2 U10: 1 NDP: 1 3PE: 1 PC1: 6	FMN: 1 MG: 1 K: 1 SF4: 6 MYR: 1 EHZ: 2 GTP: 1 CDL: 3 ZN: 1 FES: 2 U10: 1 NDP: 1 3PE: 4 PC1: 6	FMN: 1 MG: 1K: 1 SF4: 6 MYR: 1 EHZ: 2 GTP: 1 CDL: 3 ZN: 1 FES: 2 U10: 1 NDP: 1 3PE: 2 PC1: 6
B factors (Å^2)				
Protein	173.13	213.72	195.77	178.12
Ligand	194.64	216.83	208.32	193.23
R.m.s. deviations				
Bond lengths (Å)	0.007	0.007	0.004	0.007
Bond angles (°)	0.988	1.111	0.912	0.897
Validation				
MolProbity score	2.77	3.01	2.92	2.83
Clashscore	22.57	29.57	25.13	24.49
Poor rotamers (%)	4.90	5.83	5.46	4.89
Ramachandran plot				
Favored (%)	94.01	92.34	92.37	93.43
Allowed (%)	5.83	7.39	7.34	6.35
Disallowed (%)	0.16	0.27	0.29	0.22

Supplementary Table 4. Cryo-EM data collection, refinement and validation statistics

	High resolution *In-situ* structure of complex III in respiratory supercomplex (state I) (EMD-42221) (8UGD)	High resolution *In-situ* structure of complex III in respiratory supercomplex (state II) (EMD-42222) (8UGE)	High resolution *In-situ* structure of complex III in respiratory supercomplex (state III) (EMD-42223) (8UGF)	High resolution *In-situ* structure of complex III in respiratory supercomplex (state IV) (EMD-42224) (8UGG)
**Data collection and processing**				
Magnification	105,000	105,000	105,000	105,000
Voltage (kV)	300	300	300	300
Electron exposure (e–/Å^2)	50	50	50	50
Defocus range (μm)	-1.3 to -3.0	-1.3 to -3.0	-1.3 to -3.0	-1.3 to -3.0
Pixel size (Å)	0.832	0.832	0.832	0.832
Symmetry imposed	C1	C1	C1	C1
Initial particle images (no.)	16,785,436	16,785,436	16,785,436	16,785,436
Final particle images (no.)	49,704	40,836	23,075	23,877
Map resolution (Å)	3.5	3.5	3.6	3.6
FSC threshold	FSC = 0.143	FSC = 0.143	FSC = 0.143	FSC = 0.143
Map resolution range (Å)	3.2 – 4.5	3.2 – 4.5	3.2 – 4.5	3.2 – 4.5
**Refinement**				
Initial model used (PDB code)	*Ab-Initio*	*Ab-Initio*	*Ab-Initio*	*Ab-Initio*
Model resolution (Å)	3.6	3.5	3.8	3.7
FSC threshold	FSC = 0.5	FSC = 0.5	FSC = 0.5	FSC = 0.5
Map sharpening *B* factor (Å^2)	-60.7	-32.1	-19.5	-67.3
Model composition				
Non-hydrogen atoms	33536	33527	33544	33544
Protein residues	4113	4113	4113	4113
Ligands	FES: 2 HEC: 2 CDL: 6 U10: 4 PC1: 3 3PE: 11 HEM: 4	FES: 2 HEC: 2 CDL: 6 U10: 4 PC1: 3 3PE: 11 HEM: 4	FES: 2 HEC: 2 CDL: 6 U10: 4 PC1: 3 3PE: 11 HEM: 4	FES: 2 HEC: 2 CDL: 6 U10: 4 PC1: 3 3PE: 11 HEM: 4
B factors (Å^2)				
Protein	64.22	62.13	80.06	72.92
Ligand	61.54	62.05	79.56	75.23
R.m.s. deviations				
Bond lengths (Å)	0.004	0.006	0.007	0.003
Bond angles (°)	0.832	0.824	1.032	0.832
Validation				
MolProbity score	2.07	2.07	2.22	2.11
Clashscore	15.12	14.35	17.41	16.01
Poor rotamers (%)	0.75	0.95	1.12	0.72
Ramachandran plot				
Favored (%)	94.24	93.87	93.15	94.06
Allowed (%)	5.62	5.93	6.57	5.67
Disallowed (%)	0.15	0.20	0.27	0.27

Supplementary Table 5. Cryo-EM data collection, refinement and validation statistics

	In-situ complex I, Deactive _class01 (EMD-42169) (8UES)	*In-situ* complex I, Deactive _class02 (EMD-42170) (8UET)	*In-situ* complex I, Deactive _class03 (EMD-42171) (8UEU)	*In-situ* complex I, Deactive _class04 (EMD-42172) (8UEV)
**Data collection and processing**				
Magnification	105,000	105,000	105,000	105,000
Voltage (kV)	300	300	300	300
Electron exposure (e–/Å^2)	50	50	50	50
Defocus range (μm)	-1.3 to -3.0	-1.3 to -3.0	-1.3 to -3.0	-1.3 to -3.0
Pixel size (Å)	0.832	0.832	0.832	0.832
Symmetry imposed	C1	C1	C1	C1
Initial particle images (no.)	16,785,436	16,785,436	16,785,436	16,785,436
Final particle images (no.)	58,700	54,992	53,584	53,795
Map resolution (Å)	3.6	3.7	3.6	3.7
FSC threshold	FSC = 0.143	FSC = 0.143	FSC = 0.143	FSC = 0.143
Map resolution range (Å)	3.1 – 5.0	3.2 – 5.2	3.1 – 5.1	3.3 – 5.5
**Refinement**				
Initial model used (PDB code)	*Ab-Initio*	*Ab-Initio*	*Ab-Initio*	*Ab-Initio*
Model resolution (Å)	3.8	4.0	4.0	4.1
FSC threshold	FSC = 0.5	FSC = 0.5	FSC = 0.5	FSC = 0.5
Map sharpening *B* factor (Å^2)	-27.1	-27.2	-23.3	-23.6
Model composition				
Non-hydrogen atoms		67180	67263	67103
Protein residues	FMN: 1 MG: 1 K: 1 SF4:	8250	8262	8241
Ligands	6 MYR: 1 EHZ: 2 GTP: 1 CDL: 2 PGT: 1 ZN: 1 FES: 2 PC1: 5 NDP: 1 3PE: 6	FMN: 1 MG: 1 K: 1 SF4: 6 MYR: 1 EHZ: 2 GTP: 1 CDL: 2 PGT: 1 ZN: 1 FES: 2 PC1: 5 NDP: 1 3PE: 6	FMN: 1 MG: 1 K: 1 SF4: 6 MYR: 1 EHZ: 2 GTP: 1 CDL: 2 PGT: 1 ZN: 1 FES: 2 PC1: 5 NDP: 1 3PE: 6	FMN: 1 MG: 1 K: 1 SF4: 6 MYR: 1 EHZ: 2 GTP: 1 CDL: 2 PGT: 1 ZN: 1 FES: 2 PC1: 5 NDP: 1 3PE: 6
B factors (Å^2)				
Protein	124.76	107.30	107.09	116.48
Ligand	132.76	100.37	101.17	112.60
R.m.s. deviations				
Bond lengths (Å)	0.004	0.005	0.006	0.008
Bond angles (°)	0.862	0.808	0.848	1.028
Validation				
MolProbity score	2.27	2.33	2.31	2.40
Clashscore	22.86	23.17	23.16	29.63
Poor rotamers (%)	0.50	0.68	0.64	0.61
Ramachandran plot				
Favored (%)	93.56	92.31	92.89	93.02
Allowed (%)	6.27	7.43	6.89	6.75
Disallowed (%)	0.17	0.26	0.22	0.23

Supplementary Table 6. Cryo-EM data collection, refinement and validation statistics

	In-situ complex I, Deactive _class05 (EMD-42173) (8UEW)	*In-situ* complex I, Deactive _class06 (EMD-42174) (8UEX)	*In-situ* complex I, Deactive _class07 (EMD-42175) (8UEY)
**Data collection and processing**			
Magnification	105,000	105,000	105,000
Voltage (kV)	300	300	300
Electron exposure (e–/Å^2)	50	50	50
Defocus range (μm)	-1.3 to -3.0	-1.3 to -3.0	-1.3 to -3.0
Pixel size (Å)	0.832	0.832	0.832
Symmetry imposed	C1	C1	C1
Initial particle images (no.)	16,785,436	16,785,436	16,785,436
Final particle images (no.)	59,633	57,793	55,594
Map resolution (Å)	3.6	3.7	3.6
FSC threshold	FSC = 0.143	FSC = 0.143	FSC = 0.143
Map resolution range (Å)	3.2 – 5.2	3.3 – 5.5	3.1 – 5.0
**Refinement**			
Initial model used (PDB code)	*Ab-Initio*	*Ab-Initio*	*Ab-Initio*
Model resolution (Å)	3.8	4.1	4.0
FSC threshold	FSC = 0.5	FSC = 0.5	FSC = 0.5
Map sharpening *B* factor (Å^2)	-32.9	-25.2	-26.3
Model composition	67472	67180	66812
Non-hydrogen atoms	8289	8251	8202
Protein residues	FMN: 1 MG: 1 K: 1 SF4: 6 MYR: 1	FMN: 1 MG: 1 K: 1 SF4: 6 MYR: 1	FMN: 1 MG: 1 K: 1 SF4: 6 MYR: 1
Ligands	EHZ: 2 GTP: 1 CDL: 2 PGT: 1 ZN: 1	EHZ: 2 GTP: 1 CDL: 2 PGT: 1 ZN: 1	EHZ: 2 GTP: 1 CDL: 2 PGT: 1 ZN: 1
	FES: 2 PC1: 5 NDP: 1 3PE: 6	FES: 2 PC1: 5 NDP: 1 3PE: 6	FES: 2 PC1: 5 NDP: 1 3PE: 6
B factors (Å^2)			
Protein	128.11	116.12	99.22
Ligand	121.86	110.31	93.66
R.m.s. deviations			
Bond lengths (Å)	0.004	0.004	0.008
Bond angles (°)	0.810	0.810	0.978
Validation			
MolProbity score	2.26	2.33	2.35
Clashscore	22.56	22.74	25.39
Poor rotamers (%)	0.51	0.58	0.36
Ramachandran plot			
Favored (%)	93.71	92.24	92.77
Allowed (%)	6.10	7.44	7.02
Disallowed (%)	0.20	0.32	0.21

Supplementary Table 7. Isoform identification for CIV subunits.

Chain ID	Gene Name	isoform
4A	*coxI*	
4B	*coxII*	
4C	*coxIII*	
4D*	*cox4*	COX4 isoform 1
4E	*cox5A*	
4F	*cox5B*	
4G*	*cox6A*	COX6A2
4H*	*cox6B*	COX 6B1 isoform X1
4I	*cox6C*	
4J*	*cox7A*	COX 7A1
4K*	*cox7B*	COX 7B
4L	*cox7C*	
4M*	*cox8*	COX8H
4N*	*ndufa4*	NDUFA4

Supplementary Table 7 | Isoform identification for CIV subunits.

For subunits with isoforms we identify in the structure using sidechain features and list the specific isoforms in the table.

*These subunits have isoforms.

Supplementary Table 8. Analysis of "hallmarks" across different classes for combined datasets.

	Score	Q_{10}	ND3-TMH1-2 loop	NDUFS7 (47-51)	NDUFS7 (74-83)	NDUFS2-β1-β2 loop	ND1-TMH5-6 loop	ND6-TMH3	Percentage
class 0	10	No	A	loop	A	A	A	α-helix	11.90%
class 1	6.5	No	D	β-strand	$A^{§}$	A	$A^{-§}$	α-helix*	12.90%
class 2	6	No	D	loop	A^{-}	D	A^{-}	π-bulge	12.40%
class 3	5.5	No	D	β-strand	A^{-}	A^{-}	A^{-}	π-bulge	12.10%
class 4	5	No	D	β-strand	D	A	D	π-bulge	12.20%
class 5	4.5	No	$D^{§}$	β-strand	D	D	A^{-}	π-bulge	12.90%
class 6	4.5	No	D	β-strand	A^{-}	D	D	π-bulge	13.00%
class 7	4	No	D	β-strand	$D^{\#}$	D	D	π-bulge	12.40%

Supplementary Table 8 | Analysis of "hallmarks" across different classes for combined datasets (fresh, mild and harsh).

§ D denotes the confidently identified deactive state feature, while A represents the confidently identified active state feature. A^{-} indicates that the active state feature remains recognizable but shows some changes. # Arg77 and Phe76 shift and obstruct the Q_{10} channel. Although it is still recognized as an α-helix by ChimeraX, the conformation notably differs from the active state and closely resembles a π-bulge. The various states are scored based on the hallmarks. The fully active state is assigned 10 points. If it exhibited deactive features, 1 point is deducted. If it fell between the active and deactive states, 0.5 points are deducted. Class 0 represents a fully active apo state, while class 7 is categorized as the fully deactive state
