# Supplementary material for: High-resolution in situ structures of mammalian respiratory supercomplexes
Source: Nature. 2024 May 29;631(8019):232–9. doi: 10.1038/s41586-024-07488-9 (PMC11222160; doi:10.1038/s41586-024-07488-9)
Supplement: Supplementary file 2 — Reporting Summary [file 41586_2024_7488_MOESM2_ESM.pdf]

Corresponding author(s): Kai Zhang, Jiapeng Zhu

Last updated by author(s): Mar 9, 2024

Reporting Summary

Nature Portfolio wishes to improve the reproducibility of the work that we publish. This form provides structure for consistency and transparency in reporting. For further information on Nature Portfolio policies, see our Editorial Policies and the Editorial Policy Checklist.

Statistics

For all statistical analyses, confirm that the following items are present in the figure legend, table legend, main text, or Methods section.

n/a	Confirmed	
☐	☒	The exact sample size (*n*) for each experimental group/condition, given as a discrete number and unit of measurement
☐	☒	A statement on whether measurements were taken from distinct samples or whether the same sample was measured repeatedly
☒	☐	The statistical test(s) used AND whether they are one- or two-sided<br>*Only common tests should be described solely by name; describe more complex techniques in the Methods section.*
☒	☐	A description of all covariates tested
☒	☐	A description of any assumptions or corrections, such as tests of normality and adjustment for multiple comparisons
☒	☐	A full description of the statistical parameters including central tendency (e.g. means) or other basic estimates (e.g. regression coefficient) AND variation (e.g. standard deviation) or associated estimates of uncertainty (e.g. confidence intervals)
☒	☐	For null hypothesis testing, the test statistic (e.g. F, t, r) with confidence intervals, effect sizes, degrees of freedom and P value noted<br>*Give P values as exact values whenever suitable.*
☒	☐	For Bayesian analysis, information on the choice of priors and Markov chain Monte Carlo settings
☒	☐	For hierarchical and complex designs, identification of the appropriate level for tests and full reporting of outcomes
☒	☐	Estimates of effect sizes (e.g. Cohen's *d*, Pearson's *r*), indicating how they were calculated

Our web collection on statistics for biologists contains articles on many of the points above.

Software and code

Policy information about availability of computer code

Data collection	SerialEM 4.1, DigitalMicrograph 3.5. These softwares are also mentioned in the manuscript as well.
Data analysis	Relion 4.0, MotionCor2, Gctf 1.08, Gautomatch 0.60, Cryosparc 4.12, Coot 0.9.8.1, Molprobity 4.5.2, PyMOL 2.5.4, ChimeraX 1.5, Phenix 1.20.1, EMAN 2.3, IMOD 4.9.12, https://github.com/builab/subtomo2Chimera, https://github.com/JackZhang-Lab. These softwares/codes are also mentioned in the manuscript as well.

For manuscripts utilizing custom algorithms or software that are central to the research but not yet described in published literature, software must be made available to editors and reviewers. We strongly encourage code deposition in a community repository (e.g. GitHub). See the Nature Portfolio guidelines for submitting code & software for further information.

Data

Policy information about availability of data

All manuscripts must include a data availability statement. This statement should provide the following information, where applicable:

- Accession codes, unique identifiers, or web links for publicly available datasets
- A description of any restrictions on data availability
- For clinical datasets or third party data, please ensure that the statement adheres to our policy

All cryo-EM maps and atomic coordinates for mitochondrial supercomplexes have been deposited in the Electron Microscopy Data Bank (EMDB) and the Protein Data Bank(PDB), including entire and locally refined maps of type-A supercomplexes under accession codes EMD-42231/PDB-8UGP, EMD-42225/PDB-8UGH,

EMD-42226/PDB-8UGI; type-B supercomplexes under EMD-42227/PDB-8UGJ; type-O supercomplexes under EMD-42230/PDB-8UGN; type-X supercomplexes under EMD-42233/PDB-8UGR. Different classes of CI under EMD-42165/PDB-8UEO, EMD-42166/PDB-8UEP, EMD-42167/PDB-8UEQ, EMD-42168/PDB-8UER, EMD-42176/PDB-8UEZ, EMD-42169/PDB-8UES, EMD-42170/PDB-8UET, EMD-42171/PDB-8UEU, EMD-42172/PDB-8UEV, EMD-42173/PDB-8UEW, EMD-42174/PDB-8UEX, EMD-42175/PDB-8UEY. Different states of CIII under EMD-42221/PDB-8UGD, EMD-42222/PDB-8UGE, EMD-42223/PDB-8UGF, EMD-42224/PDB-8UGG, EMD-42229/PDB-8UGL. High resolution representative of complex under EMD-42143/PDB-8UD1, EMD-42228/PDB-8UGK, EMD-42229/PDB-8UGL. All these databases/datasets used in the study along with accession-codes are also mentioned in the manuscript under the "Data availability".

Research involving human participants, their data, or biological material

Policy information about studies with human participants or human data. See also policy information about sex, gender (identity/presentation), and sexual orientation and race, ethnicity and racism.

Reporting on sex and gender	N/A
Reporting on race, ethnicity, or other socially relevant groupings	N/A
Population characteristics	N/A
Recruitment	N/A
Ethics oversight	N/A

Note that full information on the approval of the study protocol must also be provided in the manuscript.

Field-specific reporting

Please select the one below that is the best fit for your research. If you are not sure, read the appropriate sections before making your selection.

☒ Life sciences ☐ Behavioural & social sciences ☐ Ecological, evolutionary & environmental sciences

For a reference copy of the document with all sections, see nature.com/documents/nr-reporting-summary-flat.pdf

Life sciences study design

All studies must disclose on these points even when the disclosure is negative.

Sample size	More than 70,000 cryo-EM movies were collected for final reconstructions. More than one million high-quality particles were included in the final classification and reconstructions of the four different types of supercomplexes. We chose this sample size to achieve the average resolution of type-A supercomplex map better than ~2.5 Å.
Data exclusions	Electron microscopy: All micrographs were checked manually, and unusable ones were excluded based on the estimated drift parameters, defocus ranges, estimated usable information by contrast transfer functions, ice thickness, 2D average qualities, 3D classification and reconstruction results using GCTF and cryoSPARC.
Replication	Multiple porcine hearts were used in the sample preparations that generated more 50 cryo-EM grids with consistent image qualities to ensure the replication. Cryo-EM structures were lowpass filtered and re-refined, which generated consistent reconstructions. All classified particles were re-processed to generate reference-free 2D class averages that are consistent multiple cycles of classification, demonstrating the replication of our classification approaches.
Randomization	All particles were randomly split for the purposes of estimating the resolutions based on the 0.143 criterion of the Fourier Shell Correlation. Otherwise randomization was not relevant to these studies.
Blinding	Blinding was not relevant to this study as no populations were preassigned to experimental groups.

Reporting for specific materials, systems and methods

We require information from authors about some types of materials, experimental systems and methods used in many studies. Here, indicate whether each material, system or method listed is relevant to your study. If you are not sure if a list item applies to your research, read the appropriate section before selecting a response.

Materials & experimental systems

n/a	Involved in the study
☒	☐ Antibodies
☒	☐ Eukaryotic cell lines
☒	☐ Palaeontology and archaeology
☒	☐ Animals and other organisms
☒	☐ Clinical data
☒	☐ Dual use research of concern
☒	☐ Plants

Methods

n/a	Involved in the study
☒	☐ ChIP-seq
☒	☐ Flow cytometry
☒	☐ MRI-based neuroimaging

Plants

Seed stocks

Report on the source of all seed stocks or other plant material used. If applicable, state the seed stock centre and catalogue number. If plant specimens were collected from the field, describe the collection location, date and sampling procedures.

Novel plant genotypes

Describe the methods by which all novel plant genotypes were produced. This includes those generated by transgenic approaches, gene editing, chemical/radiation-based mutagenesis and hybridization. For transgenic lines, describe the transformation method, the number of independent lines analyzed and the generation upon which experiments were performed. For gene-edited lines, describe the editor used, the endogenous sequence targeted for editing, the targeting guide RNA sequence (if applicable) and how the editor was applied.

Authentication

Describe any authentication procedures for each seed stock used or novel genotype generated. Describe any experiments used to assess the effect of a mutation and, where applicable, how potential secondary effects (e.g. second site T-DNA insertions, mosiacism, off-target gene editing) were examined.
